# Design of an interface for teaching cardiovascular physiology to anesthesia clinicians with a patient simulator connected to a minimally invasive cardiac output monitor (LiDCO *rapid*®)

**DOI:** 10.1186/s41077-020-00134-0

**Published:** 2020-07-31

**Authors:** Daniela Chaló, Joana Marques, Henrique Mendes, Consuelo Sancho

**Affiliations:** 1 Department of Physiology and Pharmacology, Faculty of Medicine, University of Salamanca, Salamanca, Spain; 2 Department of Anesthesiology, Centro Hospitalar do Baixo Vouga, Aveiro, Portugal; 3 SIMULA-Health Sciences School, University of Aveiro, Aveiro, Portugal; 4 Department of Anesthesiology, Hospital de Braga, Braga, Portugal; 5 SEEMSREAL, Leiria, Portugal

**Keywords:** Clinical simulation, Cardiovascular physiology, Medical education, Healthcare innovation

## Abstract

Cardiovascular physiology can be simulated in patient simulators but is limited to the simulator monitor curves and parameters, missing some important data that today is known as essential to fluid management and therapeutic decision in critical ill and high-risk surgical patients. Our main objective was to project and implement a unidirectional communication channel between a pre-existing patient simulator and a minimally invasive cardiac output monitor (LiDCO *rapid*®); a monitor that connects to real patients and interprets the arterial wave. To connect the patient simulator to the hemodynamic monitor, firstly, we had to assess both systems and design a communication channel between them. LiDCO monitor accepts as an input an analog voltage varying between 0 V and 5 V and that every volt is directly proportional to a blood pressure (mmHg) value ranging from 0 mmHg (0 V) to 500 mmHg (5 V). A Raspberry Pi 0 (Rpi0) with a WIFI chip integrated was needed and added to a digital analogue converter connected to the board. We designed a system that allowed us to collect, interpret and modify data, and feed it to the LiDCO *rapid*® monitor. We had developed a Python® script with three independent threads and a circular buffer to handle the data transmission between both systems. The LiDCO hemodynamic monitor successfully received data sent from our setup like a real patient arterial wave pulse and interpreted it to estimate several hemodynamic parameters, as cardiac output, stroke volume, systemic vascular resistance, pulse pressure variation, and stroke volume variation. The connection between the patient simulator and the LiDCO monitor is being used to create arterial curves and other hemodynamic parameters for clinical scenarios where residents and anesthesiologists can simulate a variety of unstable hemodynamic conditions, preparing them to face similar situations with real patients in a safe environment and with their own monitors.

## Introduction

Technological advances in medicine are an actual fact and the new generation of anesthesia clinicians must be prepared, since the very beginning of their residence, to use advanced technology in several equipment: monitors and patient ventilators, ultrasound and airway management devices. Also, experienced anesthesiologists need to be updated and must know how to use the new devices and how to teach to younger clinicians and residents. Active learning strategies and simulation technologies are already used with medical students [1, 2] and residents [3], and their benefits and advantages on students’ learning cognitive and behavioural skills are well recognised [4, 5]. Simulation-based learning can also be helpful to develop healthcare professional’s knowledge, skills, and attitudes while protecting patients from unnecessary risks [6]. Anesthesiologists pioneered the use of patient simulators in training programs all over the world [7, 8]. In Portugal, since 2018, the Anesthesiology Medical Council established a program with recommended courses using simulation as a teaching tool.

In recent years, significant progress has been made with perioperative technology, namely, with minimally invasive cardiac output (CO) monitors such as the LIDCO *rapid*® (LiDCO Ltd., Cambridge, UK) [9–11]. This device uses the PulseCO™ algorithm, without calibration, which converts the blood pressure arterial wave to its constituent parts of flow (cardiac output and stroke volume) and systemic vascular resistance (SVR). The PulseCO™ algorithm is scaled to each patient with a nomogram using age, height, and weight. The PulseCO™ algorithm is reliable in unstable patients and in patients on vasoactive drugs. This CO monitor also estimates: cardiac index, stroke volume index, pulse pressure variation (PPV), and stroke volume variation (SVV).

Patient simulators (PS) are an essential tool as part of the methodology in which lifelike situations are simulated and clinicians are exposed to scenarios in a safe environment which later promote self-reflection during the debriefing phase, in order to improve the clinician’s knowledge and skills. This patient simulator, METIman® Pre-Hospital (CAE Healthcare), is an advanced pathophysiological simulator that can represent different clinical scenarios, including important variations in hemodynamics, by modifying parameters such as heart contractility, aortic impedance, systemic, and pulmonary vascular resistances. Nevertheless, in most cases, advanced hemodynamic parameters, such as stroke volume, pulse pressure variation (PPV), and stroke volume variation (SVV), are not represented in the simulator’s monitors, resulting in a technological limitation. Patient simulators are also limited because of their inability to integrate with real clinical equipment, as minimally invasive cardiac output monitors.

Our main objective in this study was to project and implement an unidirectional communication channel between a pre-existing patient simulator METIman® Pre-Hospital from CAE Healthcare and a minimally invasive cardiac output monitor, LiDCO *rapid*® [12], thus being able to simulate a set of conditions on the patient simulator and make the LiDCO monitor respond to those same conditions as if a real patient was being monitored. This integration will further allow us to develop clinical scenarios and train clinicians in advanced hemodynamic monitoring using simulation.

## Material and methods

**Fig. 1:**
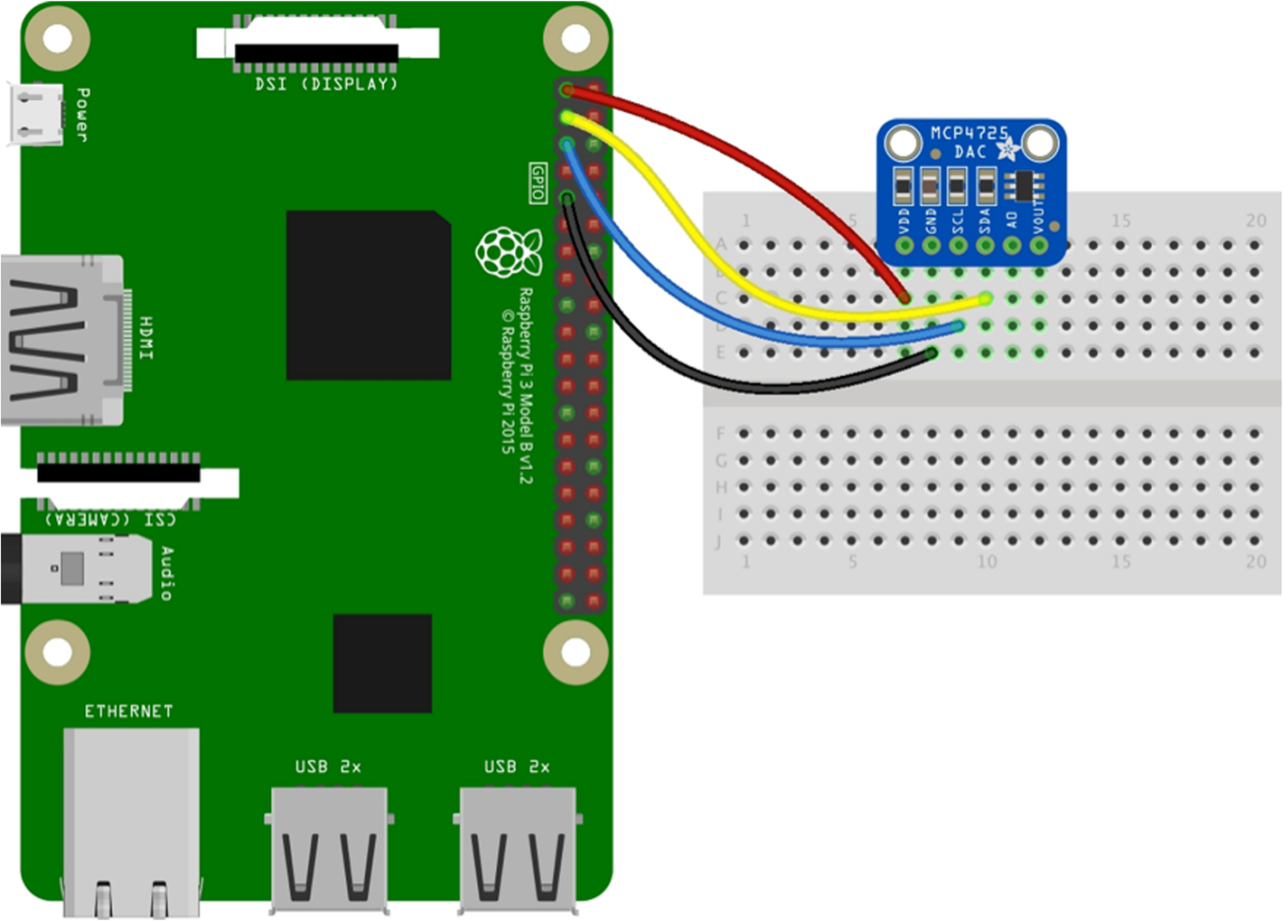
Diagram connection between the Raspberry Pi and the MCP4725 12-bit DAC

**Fig. 2:**
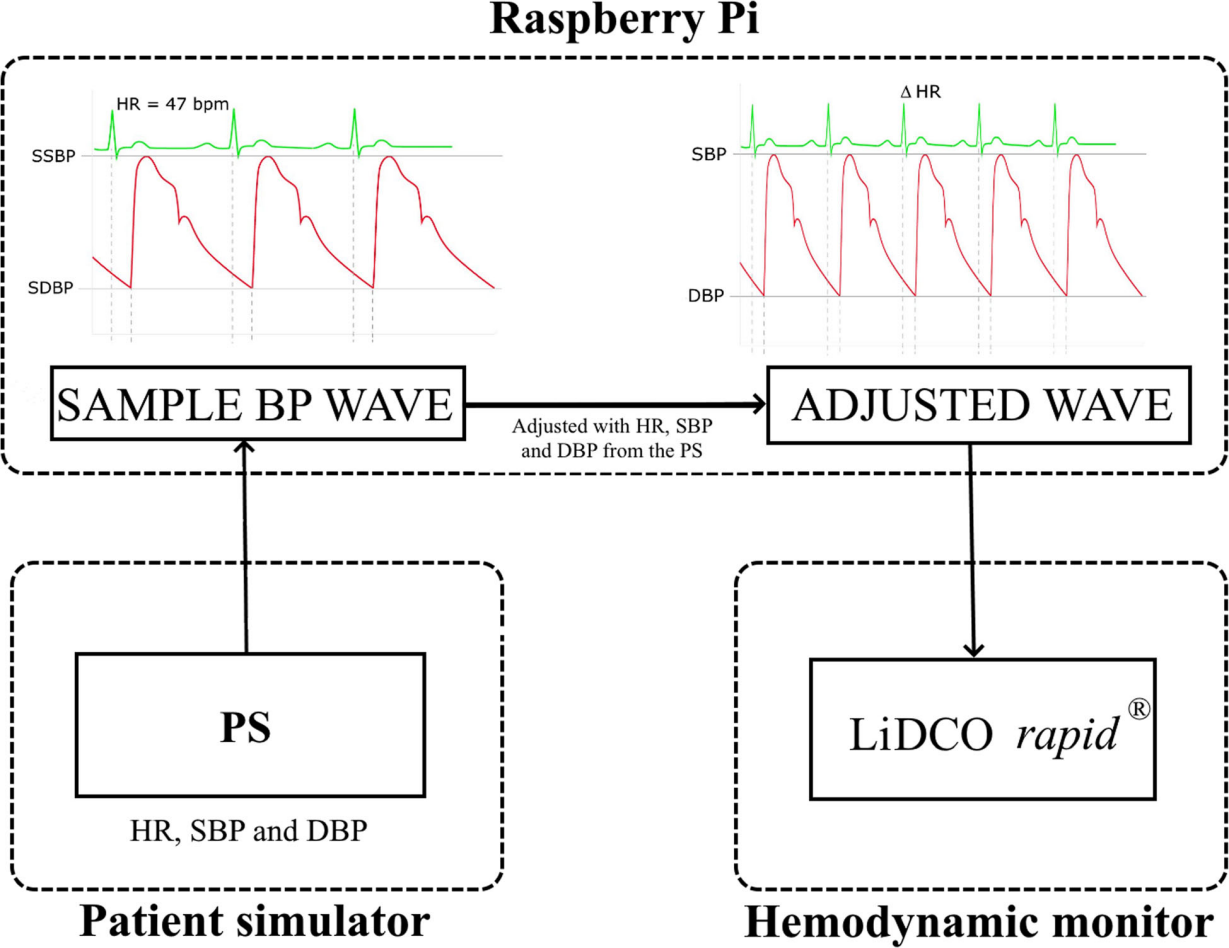
Diagram of the collecting and processing data from the patient simulator, to the Raspberry Pi and DAC, and to the hemodynamic monitor

**Fig. 3:**
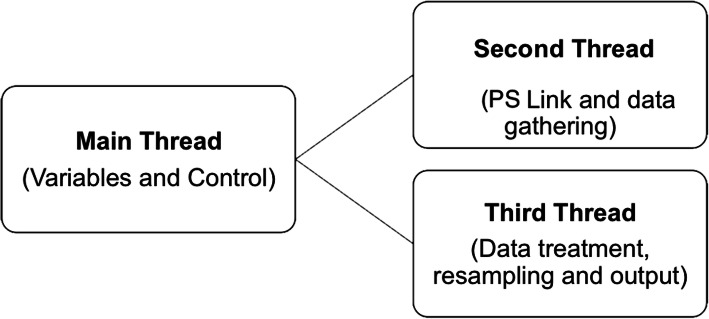
Algorithm for the connection setting with the three threads

To achieve the goal of connecting the patient simulator to the LiDCO *rapid*® monitor, firstly, we had to assess both systems and design a communication channel between them. To perform the hardware integration, we used a Raspberry Pi Zero W®. The Raspberry Pi Zero W® is a small computer on a board that runs a distribution of Linux and can be programmed on demand using Python®. We also used a DAC (digital to analogue converter) board with a MCP4725 12-bit DAC (Fig. 1) [13, 14]. This is an I2C (serial protocol for two-wire interface to connect low-speed devices like microcontrollers) controlled by DAC that can run on a 0–5 V output to generate and send a 0–5 V continuous signal to the LiDCO monitor input. For the connection between the DAC and the monitor, it used a BNC Female Jack Terminal Block compatible with the coaxial input line of the LiDCO monitor. The patient simulator data can be accessed by connecting the Raspberry Pi and the patient simulator control unit over its internal network and doing a SQL (Structured Query Language) request to its main processing unit, obtaining the systolic blood pressure (SBP), diastolic blood pressure (DBP), and the heart rate (HR), values it generates once a second. Alongside, we worked with the LiDCO development team and established that the LiDCO monitor accepts as an input an analog voltage varying between 0 V and 5 V and that every volt is directly proportional to a blood pressure (BP) (mmHg) value ranging from 0 mmHg (0 V) to 500 mmHg (5 V). Therefore, a circuit was designed to allow the Raspberry Pi to collect, interpret, and modify the data from the PS (input) and feed it into the LiDCO monitor (output) (Fig. 2). As the PS did not have a way to export the BP pulse wave values, we created an algorithm program in Python®, in which we gathered and normalized the amplitude of a standard BP pulse wave (0 mmHg–1 mmHg) from a real patient and resized it to the value range acquired from the PS (DBP/SBP). The Python® script had three independent threads and a circular buffer to handle the data transmission between both systems. The first thread (main) was responsible for setting some local variables that store function values and to initiate the two secondary threads and the circular buffer. The buffer contains the normalized array with the BP pulse wave information. The second thread connected to the PS query the HR, SBP, and DBP on 1-s intervals making it available to the third thread. The third thread received the buffer BP pulse wave values, performed the necessary calculations, and wrote the final values on the DAC (adjusted to 0–5 V) with an adjusted frequency to match the HR value on the PS (Fig. 3). Variables transmitted from the patient simulator are presented at Table 1.

**Table 1:** Output variables transmitted from the PS to the DAC

Variable	Signal type	Units	Inferior limit	Superior limit
HR	Digital (decimal)	bpm	0	350
SBP	Digital (decimal)	mmHg	NA	NA
DBP	Digital (decimal)	mmHg	NA	NA

## Results

**Fig. 4:**
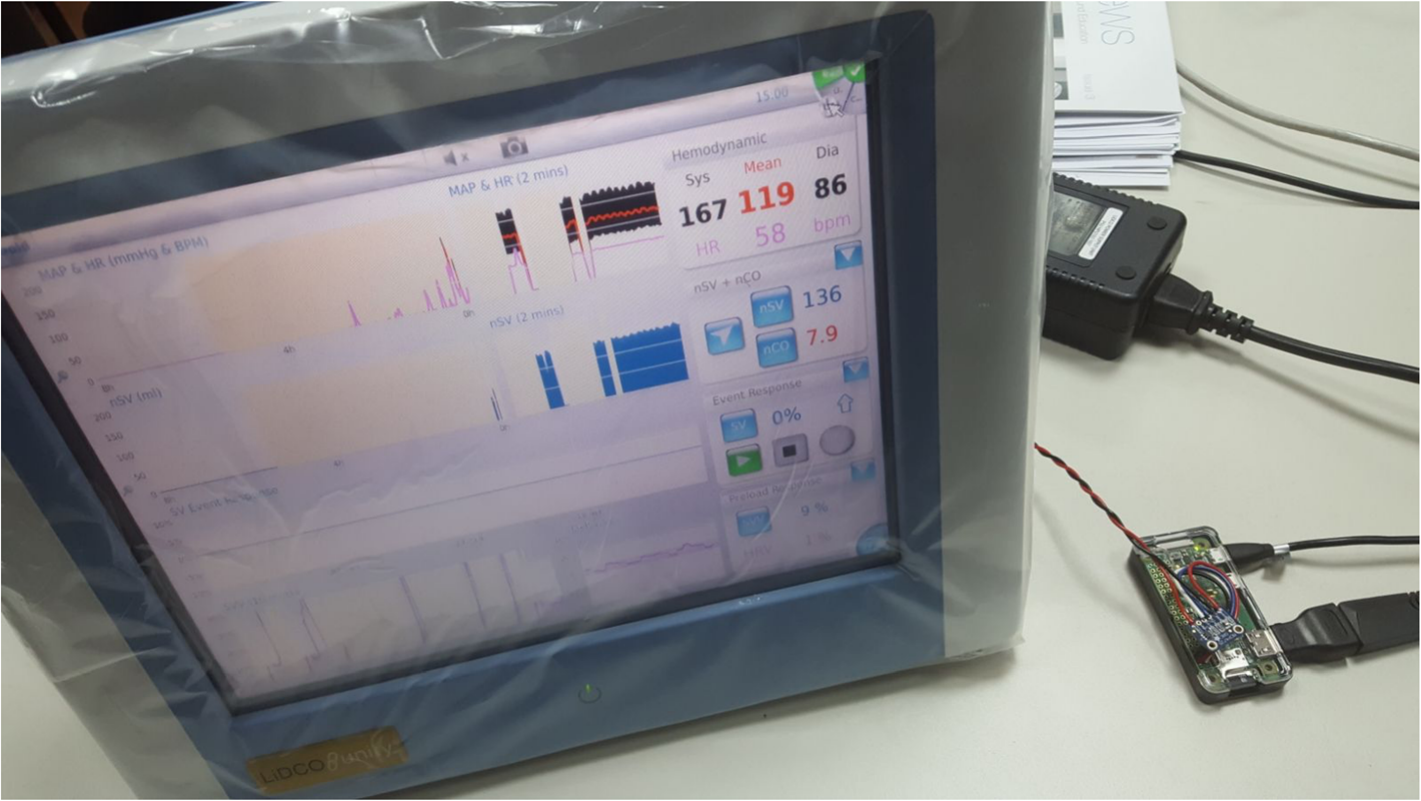
Connection between the Raspberry Pi and the LiDCO monitor

The communication between the patient simulator and the LiDCO monitor was successfully achieved. Data from the PS (HR, SBP, DBP) was extracted on a frequency of 1/s (1 Hz). This information was processed, resampled, and sent to the LiDCO monitor to be interpreted as if data from a real blood pressure pulse wave was being processed. The LiDCO hemodynamic monitor successfully received and interpreted the data sent from our setup as if it was received from a real patient, as it can be seen in the Electronic Supplementary Material (ESM.1). The interface algorithm used a normalized vector as a reference, i.e., a sample of BP waveform (signal), acquired from a patient without pathology, recorded at 250 Hz and 47 bpm (beats per minute). Then, the signal from the normalized vector was adjusted to the HR and to the BP from the PS, and the amplitude of the maximum signal and minimum signal corresponded to the SBP and to the DBP, respectively (Fig. 4).

Additional file 1: Brief video with the blood pressure pulse wave generated by the connection between the patient simulator and the LiDCO monitor

## Discussion

Cardiovascular physiology can be simulated in patient simulators but is limited to the simulator monitor curves and parameters, missing some important data essential to fluid management and goal-directed therapy (GDT) in critically ill and high-risk surgical patients. The purpose of hemodynamic monitoring is to identify variations in cardiovascular parameters and intervene before major complications occur, including organ failure or death. During surgery, fluid therapy should be targeted according to physiological measures and maintained using fluids or vasopressors once normovolemia has been established, so that tissue oxygenation would not be compromised [11, 15].

This tool is important to train not only basic cardiovascular physiology but also hemodynamic variations during anesthesia phases: induction, positioning, controlled hypotension, and other surgical conditions associated with hemodynamic compromise (orthopedic surgery, vascular surgery, major abdominal surgery) [16]. It can also be used to test enhanced recovery after surgery and emergency protocols associated with situations with hemodynamic instability like massive hemorrhage, septic shock, trauma, and obstetric hemorrhage. The implementation of GDT with the use of minimally invasive monitors to guide perioperative practice has become rapidly established and accepted over the last few years, from central venous pressure until stroke volume variation or pulse pressure variation. All minimally invasive monitors have different characteristics and layouts, so clinicians should train on their own monitor to be familiar with the parameters and its interpretation, to minimize errors and provide a better and safe healthcare.

Patients with cardiac or vascular pathology can display different hemodynamic curves, especially in those with heart diseases, abnormal contractibility, rhythm conditions, and valve-related pathologies. This fact should be taken into consideration when developing scenarios. There is a limitation related to the fact that we used a normalized vector to simulate the BP waveform, so that can only vary the amplitude of the BP wave and the HR, but not the wave configuration. Despite this limitation, the methodology still has advantages as it requests a few number of parameters from the simulator that can be, in future versions, provided from a control station, allowing the training even without the need of a patient simulator.

Authors also believe the interface could be used with other PS or other monitors. Nevertheless, this possibility is dependent on the ability to communicate with the simulator to request the three variables used (HR, SBP, and DBP). If the PS is different and the transmitted signal is analog, the approach would be easier, because the only request would be a 0–5 V analog input transmitted from the probe side.

## Conclusions

**Fig. 5:**
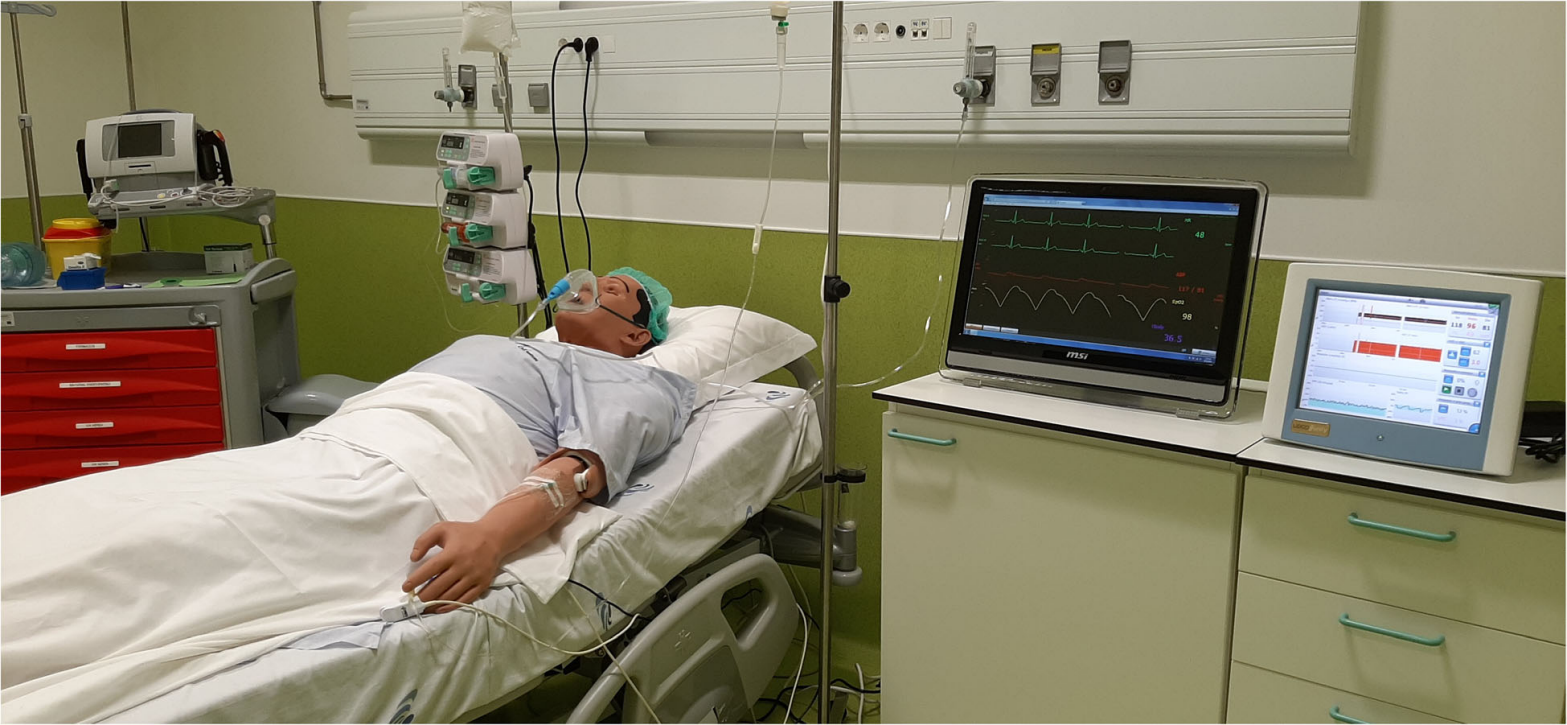
Clinical scenario in the simulation center

Anesthesiologists should be trained on their own cardiac output monitors, so they can interpret fast and easier their parameters, minimize errors, and provide a better and safe healthcare. The interface between the patient simulator and LiDCO *rapid*® monitor is now being used to teach anesthesiologists and residents with success, allowing a safe environment in a clinical simulation scenario (Fig. 5). In the near future, authors believe that the interface can be developed for other patient simulators, and it can also be used to teach other healthcare providers in an interprofessional educational program.

## Data Availability

Not applicable.

## Abbreviations

BP: Blood pressure
CO: Cardiac output
DAC: Digital to analogue converter
DBP: Diastolic blood pressure
ESM: Electronic supplementary material
GDT: Goal-directed therapy
HR: Heart rate
MAP: Mean arterial pressure
NA: Non-applicable
PS: Patient simulator
PPV: Pulse pressure variation
Rpi0: Raspberry Pi 0
SBP: Systolic blood pressure
SQL: Structured query language
SVV: Stroke volume variation
